# Combination antibiotic therapy versus monotherapy in the treatment of acute exacerbations of chronic obstructive pulmonary disease: an open-label randomized trial

**DOI:** 10.1186/s12879-021-06687-3

**Published:** 2021-09-29

**Authors:** Phuong Nguyen Thi Thu, Minh Ngo Thị Huong, Ngan Tran Thi, Hoi Nguyen Thanh, Khue Pham Minh

**Affiliations:** 1 Haiphong University of Medicine and Pharmacy, 72A Nguyen Binh Khiem, Ngo Quyen, Haiphong, Vietnam; 2 Haiphong International Hospital, 124 Nguyen Duc Canh, Le Chan, Haiphong, Vietnam; 3 Drug Administration of Vietnam, 138A Giang Vo, Ba Dinh, Ha Noi, Vietnam

**Keywords:** Antibiotic therapy, Chronic obstructive pulmonary disease, Beta-lactam antibiotics, Fluoroquinolones

## Abstract

**Background:**

The role of antibiotics in the treatment of chronic obstructive pulmonary disease (COPD) exacerbations and their effectiveness in combination have not been clearly established. To determine whether using a combination of fluoroquinolones and beta-lactams improves the clinical and microbiological efficacy of antibiotics on day 20 of treatment, we conducted an open-label randomized trial based on clinical outcomes, microbiological clearance, spirometry tests, and signs of systemic inflammation in patients hospitalized with acute exacerbations of COPD.

**Methods:**

We enrolled 139 subjects with COPD exacerbations, defined as acute worsening of respiratory symptoms leading to additional treatment. Patients were divided randomly into two groups: 79 patients using beta-lactam antibiotics alone and 60 using beta-lactam antibiotics plus fluoroquinolones. Clinical and microbiological responses, spirometry tests, symptom scores, and serum C-reactive protein (CRP) levels were evaluated.

**Results:**

Clinical success, lung function, and symptoms were similar in patients with or without fluoroquinolone administration on days 10 and 20. Combination therapy was superior in terms of microbiological outcomes and reduction in serum CRP value. Although equivalent to monotherapy in terms of clinical success, the combination showed superiority in terms of microbiological success and a decrease in CRP. The combination therapy group had a higher microbiological success rate with gram-negative bacteria than the monotherapy group with *Pseudomonas aeruginosa* (100% vs. 33.3%, respectively) and *Acinetobacter baumanii* (100% vs. 20%, respectively) (*P* < 0.05).

**Conclusions:**

Concomitant use of fluoroquinolone and beta-lactam antibiotics for bacterial infections during COPD exacerbations caused by gram-negative bacteria appear to be effective and should be applied in clinical practice.

## Background

Chronic obstructive pulmonary disease (COPD) is one of the leading causes of death in the United States and worldwide. COPD exacerbation is defined as an acute worsening of respiratory symptoms beyond one’s ability to adapt normally, requiring additional treatment and bringing a risk of adverse health events that could affect the patient in the future [1–3]. Significant progress has been made in understanding the etiology and pathogenesis of these exacerbations. In contrast, there are few clinical trials of appropriate design and quality that can support the selection of an optimal treatment approach to these exacerbations. The empirical prescription of antibiotics to treat presumed bacterial infections is common in patients presenting with acute exacerbations of COPD. However, their use is debated, and whether the choice of antibiotic is important is controversial [4, 5]. A key feature of airway inflammation in COPD is the persistent presence of bacteria in the lower respiratory tract. The most commonly isolated bacteria in the lower respiratory tract of patients with COPD are *Haemophilus influenzae*, *Moraxella catarrhalis*, and *Streptococcus pneumoniae*, with increasing evidence of the importance of *Pseudomonas aeruginosa* infection in severe COPD [6]. These bacteria exhibit high resistance to many antibiotics, and empirical treatment for serious systemic infections often involves a two-drug regimen [7, 8]. The efficacy of combination antibiotic therapy in patients with gram-negative bacillus sepsis has been previously tested, with most studies including a combination of beta-lactam antibiotics and aminoglycosides. Overall, the mortality rate for patients treated with beta-lactam-aminoglycoside combination therapy was not significantly reduced compared with that of patients with gram-negative bacillary sepsis treated with beta-lactam monotherapy [9–11]. Another potentially favorable combination is a beta-lactam plus a fluoroquinolone. However, while these combinations exhibit synergistic strength (i.e., no organisms isolated after the 24-h killing tests) due to the bactericidal activity of the combination, the minimum inhibitory concentrations of organisms recovered were unaltered [12]. With increased antibiotic resistance in gram-negative bacilli, the use of combination antibiotic therapy to treat sepsis caused by these bacilli has resurfaced [13]. Studies have shown that inappropriate antibiotic treatment can be alleviated using empirical combination therapy [14]. Despite the increasing use of fluoroquinolones due to their relatively broad spectrum of antimicrobial activity and their accepted safety, to the best of our knowledge, the inclusion of fluoroquinolones in a combined antibacterial regimen for gram-negative bacilli has not been studied on a per-drug basis.

We hypothesized that the empirical use of fluoroquinolones combined with beta-lactam antibiotics would increase their therapeutic success in patients with acute exacerbations of COPD compared with that in patients in whom beta-lactam monotherapy was used. The main goal of this study was to compare the clinical and bacterial success of a combination of beta-lactam and fluoroquinolone antibiotics with that of beta-lactam monotherapy in adult patients with COPD exacerbations.

## Methods

### Participants and study design

The study protocols were reviewed and approved by the Hai Phong International Hospital Institutional Review Board. The study was conducted in accordance with the Declaration of Helsinki and International Conference on the Harmonization of the Technical Requirements for the Registration of Pharmaceuticals for Human Use—Good Clinical Practice guidelines. All subjects gave written informed consent before study initiation. This trial was registered at ClinicalTrials.gov (NCT04879030).

The participants comprised patients aged over 45 years, diagnosed with COPD stages I–IV as stated by the Global Initiative for Chronic Obstructive Lung Disease (GOLD) [15], with acute exacerbations (onset of signs under 14 days as defined by Anthonisen et al. [16]: type 1 [increased dyspnea, increased sputum volume, and sputum purulence] or type 2 [involved two or three symptoms that needed hospitalization]), and who had used antibiotics for at least 1 day. The exclusion criteria were: recently detected or unresolved pulmonary malignancy, other infectious diseases requiring antibiotic treatment, signs of pneumonia on radiographs, and kidney failure.

### Randomization and intervention

This was an open-label, randomized study using two types of treatments. Participants were divided into two groups using a simple randomization procedure. Within 24 h of admission, patients were divided randomly into two groups by computer-generated random numbers, with one group receiving a course of single-antibiotic therapy with beta-lactam antibiotics alone and the other receiving concomitant antibiotic treatment, defined as the use of two antibiotics including one beta-lactam antibiotic and one fluoroquinolone. The beta-lactam antibiotics with activity against gram-negative bacilli in this study included piperacillin-tazobactam, ticarcillin-clavulanate, imipenem-cilastatin, meropenem, ertapenem, ceftazidime, ceftriaxone, cefotaxime, and cefixime. The fluoroquinolone antibiotics included ciprofloxacin, levofloxacin, and moxifloxacin. Other COPD medications were continued. When antibiotic therapy failed, the attending physician had the right to reevaluate the clinical status and to replace the antibiotic therapy in the study with more appropriate treatment. Safety was recorded daily with the support of a clinical pharmacist to report adverse events.

### Outcomes and follow-up

On days 1, 10, and 20, patients were evaluated clinically, and blood was drawn, collected, and the levels of C-reactive protein (CRP) were measured (Beckman Coulter, Fullerton, CA). Pulmonary function testing was performed, and expectorated sputum samples were collected by laboratory technicians. Symptoms were scored via a 10-point visual analog scale (VAS) for shortness of breath, tiredness, cough, and sputum color by a respiratory physician [17]. Separate and total scores were calculated.

The primary endpoint was the clinical outcome on day 20, as stated by Chow et al. [18]. Successful treatment was assessed as cure (completely resolved signs and symptoms related to exacerbations) or improvement (resolved or decreased symptoms and signs without new symptoms or signs related to infection) by a respiratory physician. Treatment failure was defined as the failure to address symptoms and signs, worsening of symptoms and signs, the appearance of new symptoms and signs related to the primary infection or new infection, or death.

The secondary endpoints included clinical outcome on day 10 and clinical success on days 10 and 20, based on lung function (forced expiratory volume in 1 s [FEV1]), serum CRP, symptoms, and microbiological responses.

### Microbiological response

The microbiological outcome was evaluated on days 1 and 20 in accordance with the guidelines for the clinical evaluation of anti-infective drug products by Beam et al. [19]. Success was defined as eradication (absence of the original pathogen in an adequate sputum sample after completion of therapy), presumptive eradication (clinical success in the absence of adequate sputum production), reduction (reduction of the bacterial load), or colony (the appearance of an organism that differs from that of the original pathogen in the absence of symptoms or signs of active infection). Failure was defined as persistence (continued presence of the original pathogen upon completion of therapy), relapse (the reemergence of the same pathogen after a documented absence), superinfection (emergence of a new pathogen during therapy, with associated symptoms and signs), or presumed persistence (initiation of new antimicrobial therapy for continued infection in the absence of microbiological data). The response was regarded as indeterminate in case of death or withdrawal before follow-up cultures were obtained, if microbiological data were incomplete, or if a patient had received an effective course of antibiotics that was not part of the study protocol.

### Sample size and statistical analyses

Our sample size was calculated based on the results of Ioannis et al. [20], who had a treatment success rate of 65% for the beta-lactam monotherapy group and 85% for the combination therapy group. In total, 60 exacerbations were needed in both arms of our study to compare the effect of antibiotic monotherapy and concomitant therapy on day 20 with 80% power at a significance level of 5%.

All analyses were performed using the Statistical Analysis System statistical software package, version 9.1.3 (SAS Institute, Cary, NC) and R software, version 3.2.4 [21]. Comparisons of continuous variables between two study groups were performed using a *t-*test for normally distributed data and a Mann–Whitney *U* test for non-normally distributed data; differences in frequencies of two groups were analyzed by the chi-squared test. The Fisher-exact test was used when comparing the difference in proportions of two small groups [22]. There were no missing data during the study. Subtypes were assigned depending on the type of exacerbation, presence of bacteria, serum CRP level, and pulmonary function test. Heterogeneity of therapeutic effects between subgroups was checked using logistic regression analysis.

## Results

### Baseline characteristics

**Fig. 1:**
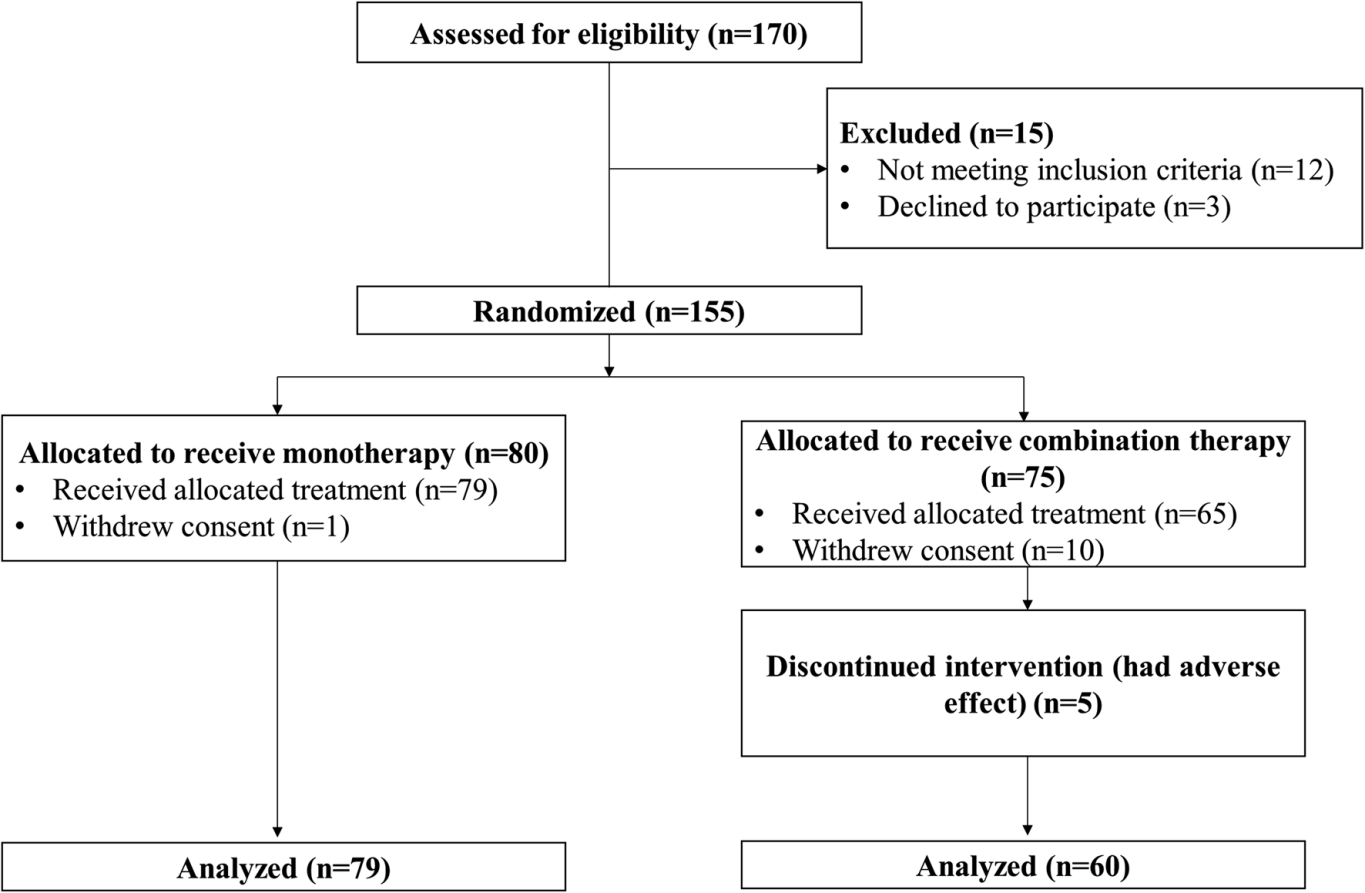
Enrollment and follow-up of patients. Shows the screening, enrollment, random assignment

Of the 170 participants screened, 155 were enrolled and randomly assigned to either the monotherapy (80 exacerbations) or combination therapy (75 exacerbations) group. Almost all participants in the monotherapy (79 of 80 [99%]) and combination groups (60 of 75 [80%]) accomplished the trial (Fig. 1). The most common cause for patients to discontinue our study was withdrawal of consent. The baseline characteristics are shown in Table 1. During the study period, the groups remained comparable with regard to demographic characteristics, smoking number, VAS score, baseline FEV1, baseline forced vital capacity, comorbidities, white blood cell count, CRP level, partial pressure of oxygen, partial pressure of carbon dioxide, inhalation corticosteroids, and systemic corticosteroid use.

**Table 1:** Baseline patient characteristics

Characteristics	Monotherapy (n = 79)	Combination therapy (n = 60)	*P*-value^#^
Age, years	71.4 ± 11.8	71.7 ± 12.1	0.89
Male sex, no. (%)	55 (69.6)	46 (76.7)	0.36
Smokers, no. (%)	43 (54.4)	34 (56.7)	0.79
Body mass index ± SD	24.1 ± 3.1	23.7 ± 2.6	0.25
VAS score	34.1 ± 11.1	32.3 ± 16.1	0.54
Percent predicted FEV1, %†	58.0 ± 14.8	55.8 ± 16.1	0.42
Percent predicted FVC, %†	71.3 ± 17.1	72.1 ± 18.2	0.47
Comorbidities, no. (%)			
Ischemic heart disease	31 (39.2)	27 (45.0)	0.52
Heart failure	20 (25.3)	13 (21.7)	0.63
Diabetes mellitus	11 (13.9)	10 (16.7)	0.46
Lung cancer	2 (2.5)	6 (10.0)	0.21
WBC			
1. > 10 × 10^9^/L, no. (%)	42 (53.1)	34 (56.7)	0.52
C-reactive protein (mg/L), median (IQR)	46.0 (12–124)	44.8 (18–113)	0.73
PaO_2_ (mm Hg), median (IQRs)	71.7 (41–99)	72.8 (43–115)	0.72
PaCO_2_ (mm Hg), median (IQRs)	42.8 (27–90)	40.9 (25–82)	0.45
ICS, no. (%)	76 (96.2)	59 (98.3)	0.08
SCS, course for current AECOPD, no. (%)	30 (37.9)	22 (36.7)	0.34

*AECOPD* acute exacerbation of COPD, *FEV1* postbronchodilator forced expiratory volume in 1 s, *ICS* inhalation corticosteroids, *IQRs* interquartile range, *SCS* systemic corticosteroids, *SD* standard deviation, *WBC* white blood cell

^#^Fisher’s Exact Test for propotion comparison; Mann–Whitney test for comparing the means

^†^Last recorded postbronchodilator value in a stable state before admission

*The body mass index is the weight in kilograms divided by the square of the height in meters

Plus–minus values represent means ± SD

### Primary outcome

Of the study population, 70 patients (88.6%) in the monotherapy group and 53 (88.3%) in the combination group (Table 2) were diagnosed as clinically successful on day 20. On day 20 of combination treatment, the clinical success was not significantly higher than that with monotherapy (*P* = 1).

**Table 2:** Effect of interventions on primary and secondary endpoints

End Point	Monotherapy (n = 79)	Combination therapy (n = 60)	*P*-value^#^
Clinical success on Day 10, no. (%)	48 (60.7)	36 (60)	1
Clinical success on Day 20, no. (%)	70(88.6)	53 (88.3)	1
FEV1, L			
FEV1 on Day 1	0.75 ± 0.19	0.73 ± 0.21	0.22
ΔFEV1 on Day 10	0.17 ± 0.21	0.20 ± 0.26	0.24
ΔFEV1 on Day 20	0.18 ± 0.28	0.24 ± 0.25	0.56
VAS score			
ΔVAS score on Day 10	− 10.6 ± 9.6	− 10.5 ± 13.3	0.92
ΔVAS score on Day 20	− 21.3 ± 15.1	− 23.8 ± 17.6	0.26
CRP (mg/L)			
ΔCRP on Day 10	− 13.5 ± 14.3	− 12.6 ± 16.1	0.099
ΔCRP on Day 20	− 22.1 ± 14.5	− 26.9 ± 15.7	0.004

*FEV1* postbronchodilator forced expiratory volume in 1 s

^#^Fisher’s Exact Test for propotion comparison; Mann–Whitney test for comparing the means

### Secondary outcomes

#### Clinical outcomes on day 10

On day 10, 48 patients (60.7%) in the monotherapy group and 36 (60%) in the combination therapy group were evaluated as clinically successful. The clinical success rates on day 10 were significantly different (*P* = 1) between the two regimens (Table 2).

#### Pulmonary function

Paired pulmonary function results were collected for 139 subjects on days 1, 10, and 20. The baseline FEV1 of monotherapy and combination therapy was 0.75 ± 0.19 L and 0.73 ± 0.21 L, respectively. There was no significant difference in FEV1 between the two therapies (*P* = 0.22) (Table 2). The mean elevation of FEV1 on day 10 was 0.17 ± 0.21 L in the monotherapy group and 0.20 ± 0.26 L in the combination therapy group (*P* = 0.24) (Table 2). On day 20, the mean elevation was 0.18 ± 0.28 L in the monotherapy group and 0.24 ± 0.25 L in the combination group (*P* = 0.56) (Table 2).

#### Serum CRP

The mean decrease in the CRP level on day 10 was − 13.5 ± 14.3 mg/L in the monotherapy group and − 12.6 ± 16.1 mg/L in the combination group (*P* = 0.099) (Table 2). On day 20, the combination group had a significantly greater decrease in CRP compared with the monotherapy group (− 26.9 ± 15.7 mg/L vs. − 22.1 ± 14.5 mg/L, *P* = 0.004).

#### Symptom scores

The mean change in total symptoms score on day 10 was − 10.6 ± 9.6 in the monotherapy group and − 10.5 ± 13.3 in the combination group (*P* = 0.92) (Table 2). On day 20, the mean change in total symptoms score was − 21.3 ± 15.1 in the monotherapy group and − 23.8 ± 17.6 in the combination group (*P* = 0.26).

#### Microbiological outcomes

We evaluated the bacteriological responses of 139 patients on days 1 and 20 (Table 3). The most predominant pathogens were *S. pneumoniae* (35.9%), *H. influenzae* (20.9%), *P. aeruginosa* (12.9%), and *Acinetobacter baumannii* (8.6%). Successful bacterial eradication was observed in 70 of the 79 patients in the monotherapy group (88.6%) and 58 of 60 (96.7%) in the combination group (*P* = 0.11) (Table 3). For the two most common pathogens (*S. pneumoniae* and *H. influenzae*), the success rates for those using combination therapy were not significantly higher than for those using monotherapy (*S. pneumoniae:* 94.4% vs. 96.9%, respectively, *P* = 1; *H. influenzae:* 87.5% vs. 95.2%, respectively, *P* = 0.48). The combination group had a higher microbiological success rate with gram-negative bacteria than the monotherapy group with *P. aeruginosa* (100% vs. 33.3%, respectively) and *A. baumanii* (100% vs. 20%, respectively) (*P* < 0.05) (Table 3).

**Table 3:** Microbiological outcomes

End Point	No. success (%)	Monotherapy (n = 79)	Combination therapy (n = 60)	*P*-value
Overall success, no. (%)	128 (92.1)	70 (88.6)	58 (96.7)	0.11
Success per pathogen				
* Streptococcus pneumoniae* (n = 50)	48	31/32 (96.9)	17/18 (94.4)	1
* Haemophilus influenzae* (n = 29)	27	20/21 (95.2)	7/8 (87.5)	0.48
* Pseudomonas aeruginosa (n* = *18)*	16	1/3 (33.3)	15/15 (100)	0.01
* Acinetobacter baumanii (n* = *12)*	8	1/5 (20.0)	7/7 (100)	0.01
* Stenotrophomonas maltophilia (n* = *10)*	9	5/6 (100)	4/4 (100)	1
* Staphylococcus aureus (n* = *8)*	8	4/4 (100)	4/4 (100)	1
* Escherichia coli (n* = *6)*	6	4/4 (100)	2/2 (100)	1
* Moraxella catarrhalis (n* = *6)*	6	4/4 (100)	2/2 (100)	1

A potential pathogen was identified in 128 exacerbations

## Discussion

There was no significant difference in clinical outcome on day 20 among patients with acute exacerbations of COPD treated with combination therapy and those treated with monotherapy. Moreover, combination therapy with fluoroquinolone was not advantageous over monotherapy for the clinical outcome on day 10 or microbiological success. However, the combination therapy group had a higher microbiological success rate with gram-negative bacteria than the monotherapy group with *P. aeruginosa* and *A. baumanii* (*P* < 0.05). The main rationale for antibiotic use in patients with COPD exacerbations has been reported to be their close association with bacteria such as *H. influenzae *[23]. Many systematic reviews have shown the benefit of antibiotics in the treatment of COPD exacerbations. Using antibiotics can help reduce recovery time, the risk of exacerbations, treatment failure, and the length of hospital stay in patients with COPD [24, 25]. Some antibiotics, such as quinolones, macrolides, beta-lactams, doxycycline, and trimethoprim-sulfamethoxazole, have proven effective in treating bacterial infections in COPD exacerbations [26]. The MOSAIC study compared broad-spectrum fluoroquinolones (such as moxifloxacin) with narrow-spectrum antibiotics (amoxicillin, clarithromycin, or cefuroxime-axetil) and found that moxifloxacin and narrow-spectrum antibiotics were comparable in terms of clinical success (improvement or cure); however, moxifloxacin was associated with a higher clinical cure rate and superior bactericidal activity [27]. Some results suggest that broad-spectrum antibiotics are more effective in treating exacerbations [16, 27]. Finally, patients using combination therapy did not show a greater decrease in symptoms on days 10 and 20 compared with those using monotherapy. There were no differences observed in the recovery of pulmonary function or resolution of main symptoms of systemic inflammation between our study groups. To the best of our knowledge, there are no studies that compare single-antibiotic and combination regimens in patients with COPD exacerbations.

Our study showed that the main causes of COPD exacerbations were *S. pneumoniae* (35.9%), *H. influenzae* (20.9%), *P. aeruginosa* (12.9%), and *A. baumannii* (8.6%). Our results are consistent with the results of other studies [28–31].

This was a randomized trial to evaluate antibiotic combination therapy for acute exacerbations of COPD. Our study did not find a significant therapeutic effect on the main outcome (clinical success on days 10 and 20). Our research findings indicate that it is unclear if there is an advantage in selecting concomitant therapy of beta-lactams and fluoroquinolone when single beta-lactam therapy can be administered for severe COPD with exacerbations, according to susceptibility testing results. However, this should be interpreted in consideration of whether the small size of our study would offer sufficient statistical power to detect large differences between the two intervention groups [32]. Moreover, this study was not placebo-controlled. Therefore, the enrollment of patients with pneumonia may have increased the observed therapeutic effect. Additionally, we selected patients with moderate-to-severe COPD with exacerbations requiring hospitalization. The severity of these exacerbations can cause a large proportion of early recurrences, thereby reducing treatment effectiveness. Since combination therapy did not outperform monotherapy in the overall analysis, it is important to evaluate whether certain subgroups benefit from antibiotics. It is clear that the airways of patients with COPD carry a certain bacterial load, and an increase in bacterial load or even the addition of a new strain of bacteria does not necessarily lead to an exacerbation [5]. In this study, we could not find any advantage in using combination therapy based on the reduction of serum CRP levels. Although serum CRP has been suggested as a biomarker of infection in acute exacerbations of COPD [33], evidence has shown that antibiotic therapy is guided by procalcitonin levels, a systemic marker of bacterial infection, which safely reduces antibiotic use [34].

The findings of this study should be explained in the context of some potential limitations. The absence of advanced antibiotic resistance in our hospital could affect the generalization of results to other studies. Another drawback of our study is that patients were not stratified depending on factors that may impact study results, such as COPD severity. However, in the subgroup evaluation, we concluded that disease severity did not affect the therapeutic outcome. Further, the VAS used in this study to score patient symptoms is not validated and has not been assessed specifically for acute exacerbations of COPD. However, the VAS is commonly used to measure subjective signs, and the VAS can reproducibly score symptoms such as shortness of breath and fatigue [35].

## Conclusion

The combination of a beta-lactam with a fluoroquinolone is equivalent to beta-lactam monotherapy in terms of clinical response on days 10 and 20 but is superior against *S. pneumoniae* and *P. aeruginosa* in terms of microbiological success on day 10. Additional effects of combination therapy include a greater reduction in the change in serum CRP levels. Further research is required before combination therapy is routinely recommended.

## Data Availability

Data available on request from the authors.

## Abbreviations

COPD: Chronic obstructive pulmonary disease
CRP: C-reactive protein
VAS: Visual analog scale
FEV1: Forced expiratory volume in 1 s
FVC: Forced vital capacity
PaO_2_: Partial pressure of oxygen
PaCO_2_: Partial pressure of carbon dioxide
